# Review of Sparse Representation-Based Classification Methods on EEG Signal Processing for Epilepsy Detection, Brain-Computer Interface and Cognitive Impairment

**DOI:** 10.3389/fnagi.2016.00172

**Published:** 2016-07-08

**Authors:** Dong Wen, Peilei Jia, Qiusheng Lian, Yanhong Zhou, Chengbiao Lu

**Affiliations:** ^1^School of Information Science and Engineering, Yanshan UniversityQinhuangdao, China; ^2^The Key Laboratory for Computer Virtual Technology and System Integration of Hebei Province, Yanshan UniversityQinhuangdao, China; ^3^School of Mathematics and Information Science and Technology, Hebei Normal University of Science and TechnologyQinhuangdao, China; ^4^School of Basic Medicine, Xinxiang Medical UniversityXinxiang, China

**Keywords:** sparse representation-based classification, sparse representation, EEG signal, preclinical mild cognitive impairment, mild cognitive impairment, Alzheimer’s disease, epilepsy, brain computer interface

## Abstract

At present, the sparse representation-based classification (SRC) has become an important approach in electroencephalograph (EEG) signal analysis, by which the data is sparsely represented on the basis of a fixed dictionary or learned dictionary and classified based on the reconstruction criteria. SRC methods have been used to analyze the EEG signals of epilepsy, cognitive impairment and brain computer interface (BCI), which made rapid progress including the improvement in computational accuracy, efficiency and robustness. However, these methods have deficiencies in real-time performance, generalization ability and the dependence of labeled sample in the analysis of the EEG signals. This mini review described the advantages and disadvantages of the SRC methods in the EEG signal analysis with the expectation that these methods can provide the better tools for analyzing EEG signals.

## Introduction

Sparse representation (SR) is used to represent data with as few atoms as possible in a given overcomplete dictionary. By using the SR, we can concisely represent the data and easily extract the valuable information from the data. The sparse representation-based classification (SRC) methods have become a research hotspot for the data processing in many fields (Vialatte et al., 2009, 2012; Liu et al., 2012; Kaleem et al., 2013; Shin et al., 2015; Yuan et al., 2015), and can greatly simplify the processing of the multi-dimensional electroencephalograph (EEG) signals from epilepsy, mild cognitive impairment (MCI), Alzheimer’s disease (AD) and brain computer interface (BCI).

Currently, studies on SRC methods used in the brain disorders and BCI involve mainly the preprocessing, SR and feature extraction, and have achieved accomplishments in computational accuracy, efficiency and robustness. Preclinical mild cognitive impairment (Pre-MCI) is a cognitive impairment status between normal aging and MCI, and also an earliest status of cognitive impairment which is more difficult to be diagnosed relative to MCI and AD (Sperling et al., 2011; Zhou et al., 2016). With the improvement of computational accuracy and efficiency, SRC methods may have potential to aid the diagnosis of Pre-MCI. However, there still exist some deficiencies needed to be solved.

**Figure 1:**
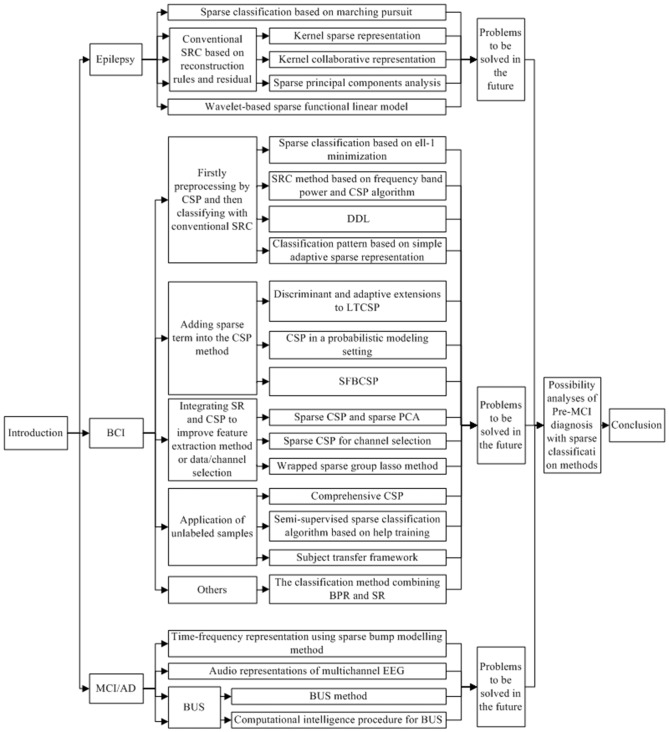
**The frame of the contents in this review.** Abbreviations: SRC, sparse representation-based classification; BCI, brain computer interface; CSP, common spatial patterns; DDL, discriminative dictionary learning; LTCSP, local temporal common spatial patterns; SFBCSP, sparse filter bands common spatial pattern; PCA, principal component analysis; BPR, biomimetic pattern recognition; SR, sparse representation; MCI, mild cognitive impairment; AD, Alzheimer’s disease; EEG, electroencephalograph; BUS, bump sonification.

This article reviewed the SRC methods in the analysis of EEG signals of epilepsy, MCI, AD and BCI, and discussed the possibility for the application of SRC methods in the diagnosis of Pre-MCI patients. The frame of this article was presented in Figure 1, and the main findings were listed in Table 1.

**Table 1:** **cognitive impairment (MCI) and Alzheimer’s disease (AD) detection using sparse representation-based classification (SRC) methods**.

**(A) Summary of studies for epilepsy detection using SRC**							

**Fields**	**Methods**	**Literatures**	**Datasets**	**Accuracy (%)**	**Sensitivity (%)**	**Specificity (%)**	**Main findings**

Epilepsy		Wang and Guo (2011)		100	100	100	Obtaining the highest accuracy and robust to noise
	Sparse classification based on marching pursuit	Guo et al. (2012)	Datasets Z (ictal) and S (healthy)	100	–	–	Reducing running time and feature dimension
		Wang et al. (2013)		100	–	–	Enhancing the classification accuracy and the efficiency simultaneously
	Kernel sparse representation	Yuan et al. (2014)	Set A (healthy), D (interictal) and E (ictal)	98.63 ± 2.80	98.25 ± 4.37	99.00 ± 4.00
	Kernel collaborative representation	Yuan et al. (2015)	Interictal and ictal iEEG database	99.99	100	100	Avoiding the choice and calculation of EEG features
	Sparse principal components analysis	Xie et al. (2012)	Set A,B (healthy) and C,D (interictal)	99.999 ± 0.0002	–	–	Focusing on the extraction of signal features with high discrimination power
	Wavelet-based sparse functional linear model	Xie and Krishnan (2013)	Set A,B (healthy), C,D (interictal) and E (ictal)	100			Effective combination of feature extraction and classification methods
			EEG data from University of Freiburg	99	–	–	

**(B) Summary of studies for BCI using SRC**							

BCI	Sparse classification based on ell-1 minimization	Shin et al. (2011)	Motor imagery (left and right hand) EEG from four healthy subjects	91.67	–	–	SRC based on ell-1 minimization
	SRC method based on frequency band power and CSP algorithm	Shin et al. (2012)	INFONET dataset from experiment and Dataset IVa from BCI competition III	75.75/96.85	–	–	SRC based on L1 minimization.
		Shin et al. (2013)	Dataset Iva from BCI competition III	96.85	–	–	Dictionary based on the CSP filtering and the band power
	Discriminative dictionary learning (DDL)	Zhou et al. (2012)	Dataset IVa from BCI competition III	70.5/94.9	–	–	Lower computational complexity and higher accuracy than SRC
	Classification pattern based on simple adaptive sparse representation	Shin et al. (2015)	Motor imagery (left and right hand, foot) EEG from 10 healthy subjects and dataset IVc from BCI Competition III	98.0/96.07	–	–	Adaptive classification techniques based on sparse representation
	Discriminant and adaptive extensions to local temporal common spatial patterns	Wang (2013)	Dataset IVa from BCI competition III and Dataset IIa of BCI competition IV	98.21/93.75	–	–	Discriminant extension: combining the between-class and the within-class scatter information. Adaptive extension: defining the weights by utilizing the sparse representation
	CSP in a probabilistic modeling setting	Wu et al. (2015)	Dataset IIIa, IVa from BCI competition III	90.68 ± 9.93	–	–	Proposing probabilistic CSP (P-CSP) model

	Sparse filter bands common spatial pattern	Zhang et al. (2015)	Dataset IVa from BCI competition III and	92.05 ± 2.45			Automatically selecting the significant filter bands to
			Dataset IIb from BCI competition IV	81.17 ± 3.55	–	–	improve classification performance
	Sparse CSP and sparse PC A	Shi et al. (2011)	Dataset IIIa from BCI competition III	90	–	–	Sparse subspace learning technique
	Sparse CSP for channel selection	Arvaneh et al. (2011)	Dataset IIa from BCI competition IV and	82.55 ± 12.8			Improving performance in the case of noise
			Dataset IVa from BCI competition III	73.5 ± 15.1	–	–	interference and limited data
		Goksu et al. (2011, 2013)	ECoG dataset of BCI competition 2005	90	–	–	Extension of the greedy search based solution to multiple sparse filters
		Tomida et al. (2015)	Dataset IVa from BCI competition III and Dataset I from BCI competition IV	87.64/81.25	–	–	Introducing weighted averaging with weight coefficients rejecting low quality trials
	Wrapped sparse group lasso method	Wang et al. (2015)	Dataset I from BCI competition IV	84.72	–	–	Simultaneously achieving channel and feature selection with a lower error rate

	Comprehensive CSP	Wang and Xu (2012)	Dataset IVa from BCI competition III and EEG motor movement/ Imagery dataset	98.2/89.5	–	–	Contributing a comprehensive CSP (cCSP) that learns on both labeled and unlabeled trials
	Semi-supervised sparse classification Jiaetal., algorithm based on 2014 help training	Jia et al. (2014)	Dataset I from BCI competition I and Dataset II-IV from BCI competition II	97/82	–	–	Selecting samples with high confidence according to sparse representation classifier
	Subject transfer framework	Tu and Sun (2012)	EEG data from the NIPS 2001 BCI workshop	72.49	–	–	Reducing the training sessions of the target subject by utilizing samples from other subjects
	The classification method combining BPR and SR	Ge and Wu (2012)	Dataset 1: Iva in 2005 BCIC III	94	–	–
		Ren et al. (2014)	Dataset 1: Iva in 2005 BCIC III	97			Utilizing SR to solve the overlapping coverage problem of BPR
			Dataset 2: from Tongji University	82.6	–	–
			Dataset 3: from BCIC III	88.03

**(C) Summary of studies for MCI/AD detection using SRC**							

MCI/AD	Time-frequency representation using sparse bump modeling method	Vialatte et al. (2005a,b)	Healthy control, MCI patients and AD patients	93	86.4	97.4	Compressing nformation contained in EEG time- frequency maps
	Bump sonification (BUS) method	Vialatte and Cichocki (2006)		–	–	–	Perceiving simultaneously every channel, and analyzing more tractably the time dynamics of the signals

	Computational intelligence procedure for BUS	Vialatte et al. (2009)	Mildly impaired patients progression towards AD group and Control group	89	–	–	Applicating BUS to online sonification
	Audio representations of multichannel EEG	Vialatte et al. (2012)	MCI group and Control group	89	–	–	Presenting a physiologically inspired method for generating music scores from multi-channel EEG

## The EEG Signal Analysis Methods Based on SRC

### Application and Performance Evaluation of SRC in Epilepsy Detection

#### Method Description and Evaluation

Currently, there are three perspectives of SRC methods used in epileptic detection, including reconstruction rules and residual error classifications on the whole classification stage, overcomplete dictionary on the preprocessing stage, and wavelet-based sparse functional linear model on the feature extraction stage.

For the first perspective, as the reconstruction rule classifications do not need to extract features or to design a classifier, the applied range of the methods is therefore greatly improved, and is superior than the traditional epilepsy detection methods. Using the classification method based on kernel SR and kernel collaborative representation, the classification accuracy in analyzing the epilepsy EEG signals reached up to 98.63% and 99.99% respectively, and the fast speed in computation can help to monitor epilepsy in real-time (Yuan et al., 2014, 2015).

Using above methods, good performance in classifications were demonstrated between epileptic patients with ictal EEG normal control group, or between epileptic patients with interictal EEG and ictal EEG However, for the classification between epileptic patients with interictal EEG and normal control group, whether these methods can achieve the similar performance remains to be further verified. Recently, using sparse principal components analysis method with reconstruction rules, the performance of classification between epilepsy patients with interictal EEG and normal control group was demonstrated to be excellent (Xie et al., 2012; Xie and Krishnan, 2013).

For the second perspective, Wang and Guo (2011) initially proposed SR based on matching pursuit and selected decomposition coefficients and atom parameters as features. However, the computation complexity was relatively high. To reduce the computation complexity, they then proposed Harmony Search method to find the optimal atom parameters, and selected the decomposition coefficients, FR parameters and restructured error to constitute a feature vector (Guo et al., 2012). By constituting the feature vector using decomposition coefficients, atom parameters and FR parameters, the computing time was further reduced (Wang et al., 2013).

For the third perspective, using wavelet-based sparse functional linear model, the accuracy in classifying epilepsy patients with interictal EEG from normal control group was up to 100% (Xie et al., 2012; Xie and Krishnan, 2013). However, the computation efficiency of feature extraction needs to be improved by using the methods, such as signal decomposition algorithms (Kaleem et al., 2013).

#### Problems to be Solved in the Future

A first problem is how to automatically determine the appropriate dictionary size and feature number of overcomplete dictionary. Secondly, the computation speed of SR needs to be improved. The aspects of the improvement may include dictionary learning algorithm and sparse coefficient solution algorithm. Thirdly, the difference between epileptic patients with interictal EEG and normal control group need to be analyzed in depth. It is the main reason why actual performance of different methods can be distinguished only when the difference between two kinds of signals is very small.

### Application and Performance Evaluation of SRC in BCI

#### Method Description and Evaluation

Five perspectives of the SRC methods applied in BCI system were presented in this review. The main stream idea of the first three perspectives is to improve the classification performance, the feature extraction and data selection by combining SR with common spatial patterns (CSP). For the fourth perspective, researchers used unlabeled samples to improve the classification performance. As for the fifth perspective, some scholars proposed integrating SR with other traditional classification methods.

For the first perspective, some researchers used CSP and conventional SRC methods for signal preprocessing and classifying, respectively. SRC method based on ell-1 minimization has a classification accuracy of 91.67% (Shin et al., 2011), and the classification accuracy in constructing dictionary reached 96.85% when using the band power feature of signal filtered by CSP (Shin et al., 2012, 2013). However, it is difficult to select the appropriate number of CSP filters, and the computation complexity still needs to be reduced. In view of this, recently proposed discriminative dictionary learning (DDL) improved the classification accuracy and computational efficiency (Zhou et al., 2012). A new classification method based on simple adaptive SR also showed a relatively high classification accuracy (Shin et al., 2015).

For the second perspective, sparse term is often used to improve the performance of the CSP method. Wang (2013) integrated discriminant and adaptive extensions to local temporal CSP, which had better classification accuracy. CSP algorithm was cast in a probabilistic modeling setting to overcome overfitting problem of CSP by using of sparse Bayesian learning (Wu et al., 2015). Sparse filter bands common spatial pattern (SFBCSP) recently proposed by Zhang et al. (2015). showed an improved classification accuracy. However, the determination of the regularization parameter *λ* in SFBCSP is time consuming, and SFBCSP is not suitable for the analysis of the data set with small samples.

For the third perspective, SR and CSP are often integrated to improve the effectiveness of feature extraction or data/channel selection. In respect of feature extraction, the sparse component analysis (SCA) and CSP were utilized to construct a combined feature vector (Li et al., 2005). The sparse CSP and sparse principal component analysis (PCA) were applied to select relevant EEG components and extract EEG features in BCI system, respectively (Shi et al., 2011). However, there exists a vast improvement space in the classification accuracy of these methods.

The classification performance can be improved according to the selection of different data/channels. A sparsity-aware method was proposed in order to select and remove low-quality trial data (Tomida et al., 2015). When applying L1 regularization term to CSP, Yong et al. (2008) showed that the average number of electrodes was reduced to 11% with a slight decrease of classification accuracy. To ensure the lowest reduction degree of classification performance, the minimal subset of EEG channels was selected for the classification. When L1/L2 norm was combined with CSP, the performance of channel selection algorithm was improved in the case of noise interference and limited data (Arvaneh et al., 2011). A sparse CSP (sCSP) method proposed by Goksu et al. (2011, 2013) showed a low computation complexity. However, the performance may be decreased when the different samples were used or the number of training samples is low.

A wrapped sparse group lasso method to select mixed EEG channel feature is suitable for high dimensional feature fusion. Stability and computing speed in this method were high, but the classification accuracy needs to be improved (Wang et al., 2015). The channel selection methods with CSP likely were trapped in a local minimum due to the non convexity of the optimization problem in CSP, which resulted in a decline in classification accuracy (Goksu et al., 2013).

For the fourth perspective, the less training samples will lead to the generalization performance deterioration caused by over-fitting, and it is easy to obtain unlabeled samples. Therefore, some researchers studied comprehensive learning mode to combine the labeled with unlabeled data, and showed that the classification performance was largely improved compared to the traditional CSP. The comprehensive learning mode includes the comprehensive CSP and semi-supervised SRC algorithm (Wang and Xu, 2012; Jia et al., 2014). A subject transfer framework reduced the training sessions of the target subjects by utilizing samples from other subjects and improved the classification accuracy (Tu and Sun, 2012). However, the computation complexity of this method was high, and the number of samples must be equal, which limited its application in reality.

For the fifth perspective, biomimetic pattern recognition (BPR) and SR were combined to accomplish the task of classification (Ge and Wu, 2012). A new classification method which combined BPR and SR under the semi-supervised co-training framework was recently proposed (Ren et al., 2014). These methods utilized SR to solve the overlapping coverage problem of BPR, and the classification accuracy was greatly increased compared to traditional classification methods. Mixed alternating least squares based on nonnegative matrix factorization were proposed to analyze event-related potential and event related spectral perturbation features. As a consequence, the performance of the algorithm was increased (Sburlea et al., 2015).

#### Problems to be Solved in the Future

Some problems remain to be solved in the field of BCI application. On account of channel selection in SRC, it is necessary not only to reduce channels, but also to maintain a high classification rate at the same time. Nevertheless, how to balance both is a challenge. It is still a research focus to determine the appropriate number of spatial filters in order to avoid over-fitting and meet the requirements of sparse coefficient solution. In addition to the principle based on the minimization of the reconstruction error, it is necessary to select new perspectives in the dictionary construction methods.

### Application and Performance Evaluation of SRC in Detection of MCI and AD

#### Method Description and Evaluation

There are a few studies about SRC methods for the detection of MCI and AD. Most studies focused on the angle of sparse bump modeling. The classification accuracy was 93% when using the sparse bump modeling method in the analysis of the EEG signal (Vialatte et al., 2005a,b). However, it still needs validation with more datasets. A BUS method (Vialatte and Cichocki, 2006) and a computational intelligence procedure for online sonification were proposed by Vialatte et al. (2009, 2012). The results showed high identification accuracy and also confirmed the potential of these methods to be used in real-time diagnosis. In Vialatte et al. (2011) improved the classification specificity of clinical EEG by means of wavelet transform and sparse bump modeling. However, the application of sparse bump modeling method is limited to the analysis of the events at low frequency bands. And for the reason of using a low pass filter, gamma band activity did not suitable for the analysis in using this method.

#### Problems to be Solved in the Future

When utilizing SRC for the analysis of EEG signals from MCI and AD patients, the classification performance of SRC can be improved by using sparse Bayesian learning method to extract coupling and synchronization feature. For the MCI classification, the space sparsity of the brain areas and time sparsity of channel samples need to be considered. Reducing the amount of data participating in the classification by selecting channels will promote the classification performance.

### Application of SRC Methods for the Analysis of EEG Signal of Pre-MCI Patients

Application of SRC method in the analysis of epilepsy, BCI, MCI and AD has achieved considerable achievements, however no relevant research literatures about Pre-MCI diagnosis using SRC methods can be found. We thus proposed to use SRC method for Pre-MCI diagnosis. The small difference in EEG signals between the Pre-MCI patients and normal control group makes the diagnosis of Pre-MCI difficult. However, if the accuracy, sensitivity, specificity and computing speed of SRC methods can be further improved, it is possible that these methods can be used for the diagnosis of the Pre-MCI. As the data dimension of Pre-MCI is high, we need to consider the space sparsity of brain areas and time sparsity of EEG signals of every channel, reduce the amount of data used in the classification by selecting channels in order to improve classification performance and enhance the effectiveness in the dictionary design, feature extraction and SR.

## Conclusion

We evaluated the SRC methods in the analysis of EEG signals from epilepsy, BCI, MCI and AD and illustrated the characteristics, advantages and disadvantages of various methods. The SRC methods have become an effective tool in aiding the diagnosis of brain disorder. Further improving the current SRC methods by such as combining SR with CSP will largely increase the classification accuracy and efficiency as well as sensitivity, making it potential for the application in diagnosis of Pre-MCI.

## Author Contributions

DW and YZ designed the study and wrote this article. PJ and QL wrote this article. CL designed the study and revised this article.

## Conflict of Interest Statement

The authors declare that the research was conducted in the absence of any commercial or financial relationships that could be construed as a potential conflict of interest.
